# Cerebrospinal fluid shunt surgery reduces the risk of developing dementia and Alzheimer’s disease in patients with idiopathic normal pressure hydrocephalus: a nationwide population-based propensity-weighted cohort study

**DOI:** 10.1186/s12987-024-00517-9

**Published:** 2024-02-14

**Authors:** Pao-Hui Tseng, Wan-Ting Huang, Jen-Hung Wang, Bor-Ren Huang, Hsin-Yi Huang, Sheng-Tzung Tsai

**Affiliations:** 1 Department of Neurosurgery, Hualien Tzu Chi Hospital, Buddhist Tzu Chi Medical Foundation, Hualien, 970 Taiwan; 2 Department of Nursing, Hualien Tzu Chi Hospital, Buddhist Tzu Chi Medical Foundation, Hualien, 970 Taiwan; 3 Institute of Medical Sciences, Tzu Chi University, Hualien, 970 Taiwan; 4 Department of Medical Research, Hualien Tzu Chi Hospital, Buddhist Tzu Chi Medical Foundation, 707, Sec 3, Zhongyang Road, Hualien, 970 Taiwan; 5 Department of Neurosurgery, Taichung Tzu Chi Hospital, Buddhist Tzu Chi Medical Foundation, Taichung, 427 Taiwan; 6 School of Medicine, Tzu Chi University, Hualien, 970 Taiwan

**Keywords:** Idiopathic normal-pressure hydrocephalus, Cerebrospinal fluid shunt surgery, Dementia, Alzheimer’s disease, Vascular dementia

## Abstract

**Background:**

Patients with idiopathic normal-pressure hydrocephalus (iNPH) are predisposed to developing dementing disorders. Cerebrospinal fluid (CSF) shunt implantation is a treatment used to improve the motor and cognitive disabilities of these patients; however, its effect on the risk of developing dementing disorders remains unclear. We conducted a population-based propensity-weighted cohort study to investigate whether CSF shunt surgery may reduce the risk of subsequently developing dementia, Alzheimer’s disease (AD), and vascular dementia in iNPH patients.

**Methods:**

Patients aged ≥ 60 years who were diagnosed with iNPH (n = 2053) between January 2001 and June 2018 were identified from the Taiwan National Health Insurance Research Database. Various demographic characteristics (age, sex, and monthly income) and clinical data (incidence year, comorbidities, and Charlson comorbidity index) were collected and divided into the shunt surgery group (SSG) and the non-shunt surgery group (NSSG). Stabilized inverse probability of treatment weighting by using the propensity score was performed to achieve a balanced distribution of confounders across the two study groups. The cumulative incidence rate and risk of dementing disorders were estimated during a 16-year follow-up period.

**Results:**

After weighting, the data of 375.0 patients in SSG and 1677.4 patients in NSSG were analyzed. Kaplan–Meier curve analysis indicated that the cumulative incidence rate of AD (*p* = 0.009), but not dementia (*p* = 0.241) and vascular dementia (*p* = 0.761), in SSG was significantly lower than that in NSSG over the 16-year follow-up period. Cox proportional hazards regression analysis revealed that SSG had a reduced hazard ratio (HR) for developing AD [HR (95% CI) 0.17 (0.04–0.69)], but not for dementia [HR (95% CI) 0.83 (0.61–1.12)] and vascular dementia [HR (95% CI) 1.18 (0.44–3.16)], compared with NSSG. Further Fine–Gray hazard regression analysis with death as a competing event demonstrated that SSG had a reduced subdistribution HR (sHR) for developing dementia [sHR (95% CI) 0.74 (0.55–0.99)] and AD [sHR (95% CI) 0.15 (0.04–0.61)], but not for vascular dementia [sHR (95% CI) 1.07 (0.40–2.86)].

**Conclusion:**

CSF shunt surgery is associated with reduced risks of the subsequent development of dementia and AD in iNPH patients. Our findings may provide valuable information for assessing the benefit-to-risk profile of CSF shunt surgery.

**Supplementary Information:**

The online version contains supplementary material available at 10.1186/s12987-024-00517-9.

## Introduction

Idiopathic normal-pressure hydrocephalus (iNPH) is a potentially treatable neurological disorder among the elderly population; it is characterized clinically by dilated cerebral ventricles and the triad of gait disturbance, urinary incontinence, and cognitive deficit without any recognized cause [1–3] The incidence of iNPH ranges from 10 per 100,000 to 22 per 100,000 overall, with 1.30% among those aged ≥ 65 years and 5.9% among those aged ≥ 80 years [4, 5]. Although the pathogenesis of iNPH remains largely unclear, communicating hydrocephalus resulting from an excess accumulation of cerebrospinal fluid (CSF) due to an imbalance between its formation and removal is generally believed to initiate a vicious cycle of neurological damages in iNPH [3]. Guidelines recommend CSF shunt implantation, including ventriculoperitoneal and lumboperitoneal shunts, as treatment for improving motor (gait disturbance and urinary incontinence) and cognitive disabilities among iNPH patients [6, 7]. Several studies have demonstrated that CSF shunt surgery can improve these outcomes in iNPH patients [1, 6–10]. However, not all iNPH patients undergo CSF shunt surgery [1, 6, 7, 10, 11], and the decision is typically based on the conjoined input of the neurologist, patient, and his/her family members after a full discussion and assessment of its risks and benefits [12].

Several studies [13–15] have reported that 21%–73% of non-shunted or shunted iNPH patients developed Alzheimer’s disease (AD) or vascular dementia during a median follow-up period of 4.8–5.3 years. The analysis of biomarkers by using brain biopsies obtained during CSF shunt surgery revealed a high prevalence of typical AD histological findings, suggesting pre-stage AD in iNPH patients [16, 17]. A report from the task force of the International Society for Hydrocephalus and Cerebrospinal Fluid Disorders (ISHCSF) [18] proposed that iNPH may increase the risk of AD and other dementing disorders. iNPH and AD have been hypothesized to have a common physiological basis in CSF circulatory dysfunction and failure, and the risk of AD may increase once iNPH takes hold [19]. When taken together, these studies suggest that shunted and non-shunted iNPH patients are predisposed to developing dementing disorders. However, the effect of CSF shunt surgery on the risk of developing dementing disorders among iNPH patients remains unclear.

The objective of the current study was to investigate whether CSF shunt surgery may reduce the risk of developing dementing disorders, including dementia, AD, and vascular dementia, among iNPH patients over time. We used a nationwide population-based dataset to conduct a propensity-weighted cohort study to achieve this goal. Patients were classified into the shunt surgery group (SSG) and the non-shunt surgery group (NSSG) for the subsequent risk analysis.

## Methods

### Data sources

A population-based propensity-weighted cohort study was conducted using the Taiwan National Health Insurance Research Database (NHIRD) from 2000 to 2018. The National Health Insurance program provides the entire population of Taiwan with universal health insurance and covers 99% of their healthcare needs and medical services. NHIRD has detailed medical records of each patient, including information about basic demographic variables, outpatient visits, patient admissions, surgical procedures, medication prescriptions, and health providers [20]. In the current study, we used the de-identified dataset that comprised 2 million subjects who were randomly selected from the list of National Health Insurance beneficiaries. This de-identified dataset was released by the Data Science Center of the Ministry of Health and Welfare of Taiwan for approved research projects [20, 21]. The collected disease diagnostic codes were based on coding systems, including the codes of the International Classification of Diseases, Ninth Revision, Clinical Modification (ICD-9-CM) and its Tenth Revision (ICD-10-CM) (Additional file 1: Table S1). Their validity and accuracy have been demonstrated in previous studies [20, 21]. Moreover, procedure and Anatomical Therapeutic Chemical (ATC) codes were used to identify types of surgery and drug prescriptions, respectively (Additional file 1: Table S1). Patients with iNPH, dementia, AD, and vascular dementia were identified using diagnostic codes. ATC codes were additionally used to identify patients with dementia, while procedure codes were used to identify patients with CSF shunt surgery. This study was approved by the Institutional Review Board of Hualien Tzu Chi Hospital (IRB110-161-C). For this retrospective study, informed consent was waived in accordance with the institutional guidelines.

### Study cohort and design

**Fig. 1:**
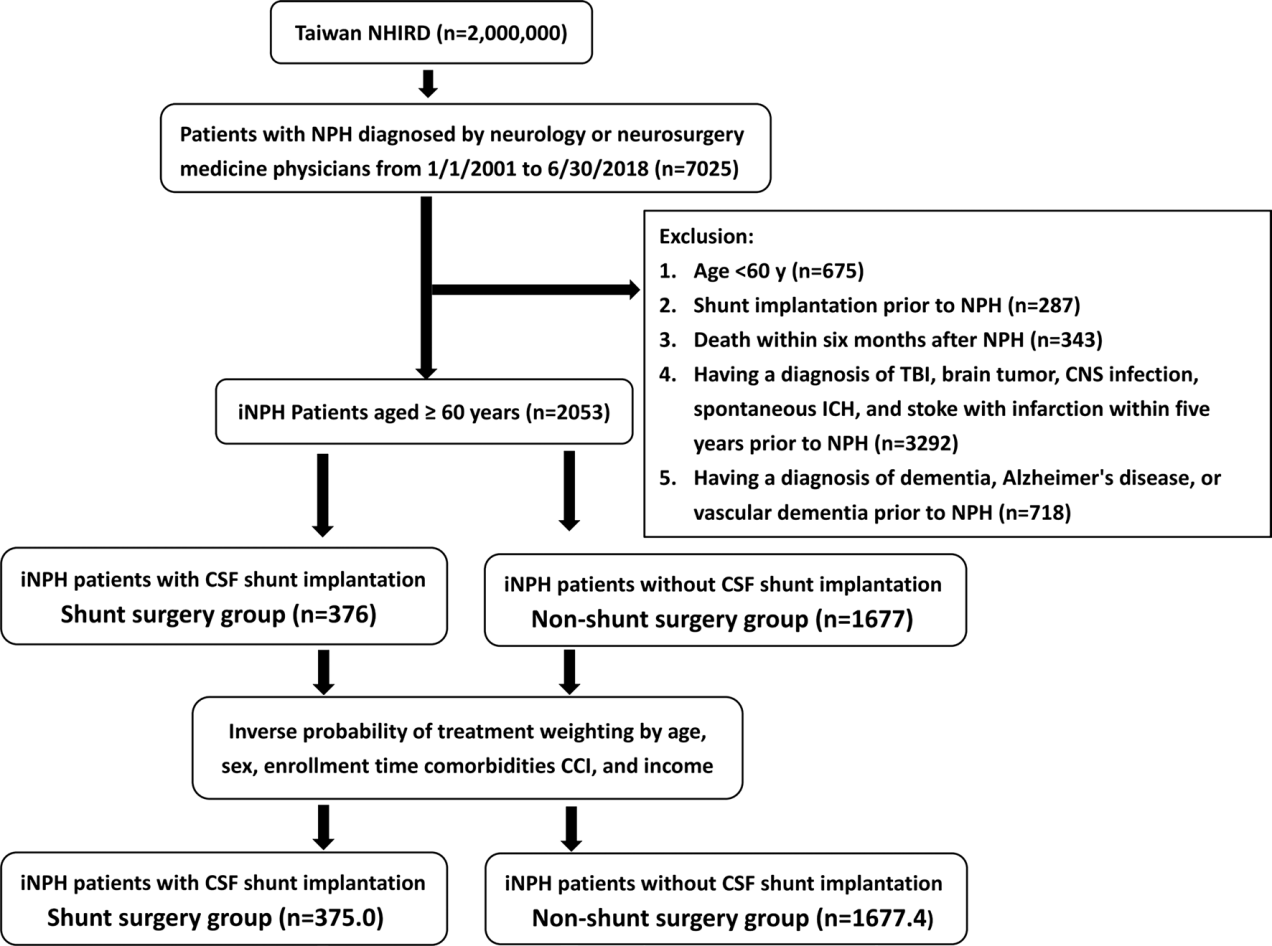
Patient selection flowchart. NHIRD, National Health Insurance Research Database. iNPH, idiopathic normal-pressure hydrocephalus. CSF, cerebrospinal fluid. TBI, traumatic brain injury. CNS, central nervous system. ICH, intracerebral hemorrhage. CCI, Charlson comorbidity index

We searched the NHIRD records from January 2001 to June 2018 to identify patients diagnosed with normal-pressure hydrocephalus (NPH) (Fig. 1). The exclusion criteria were as follows: (1) patients aged < 60 years; (2) patients with CSF shunt implantation prior to the diagnosis of NPH; (3) patients who died within 6 months after the diagnosis of NPH; (4) patients diagnosed with traumatic brain injury, brain tumor, central nervous system infection, spontaneous intracerebral hemorrhage, or stroke with infarction within 5 years prior to the diagnosis of NPH; and (5) patients diagnosed with dementia, AD, or vascular dementia prior to the diagnosis of NPH. NPH patients were included only if they had one record of hospitalization or at least two records of outpatient visits with the diagnosis as suggested by a previous study [22]. In Taiwan, the diagnosis of NPH usually was made by neurologists or neurosurgeons based on the recommendations from the Japanese Guidelines [23, 24], which were available during the study period. The diagnosis usually was made based on clinical symptoms, radiological findings, and findings from CSF drainage tests. The diagnosis of dementia, AD, or vascular dementia usually was made based on the recommendations from the relevant Guidelines [25–27]. After exclusion, the included subjects were defined as iNPH patients aged ≥ 60 years (Fig. 1). These iNPH patients were then classified into two study groups. One group (SSG) received CSF shunting placement surgery within 6 months after being diagnosed with iNPH, whereas the other group (NSSG) did not receive CSF shunting placement surgery. The index date was set as 1 year after the diagnosis date of iNPH. The follow-up period was initiated from the index date and ended with either the occurrence of the event, death, withdrawal from the insurance system, or December 31, 2018, whichever came first.

### Covariates and outcomes

Basic demographic data, including age, sex, and monthly income, were collected. Comorbidities, including coronary artery disease, chronic kidney disease, hypertension, and type II diabetes mellitus, were identified through the corresponding ICD-9-CM and ICD-10-CM codes (Additional file 1: Table S1) and used to calculate the Charlson comorbidity index (CCI). The outcomes of this study were the events of dementing disorders, including dementia, AD, and vascular dementia, occurring during the follow-up period identified by the corresponding ICD-9-CM, ICD-10-CM, and ATC codes (Additional file 1: Table S1).

### Statistical analysis

Continuous variables were presented as mean ± standard deviation. Categorical variables were presented as frequencies and percentages. To reduce bias due to confounding factors, we calculated propensity scores to estimate the probability of patients being assigned to SSG and NSSG via a logistic regression model. The model included age, sex, income level, incidence year, CCI, and comorbidities. Stabilized inverse probability of treatment weighting (IPTW) [28, 29] by using the propensity score was then performed to create a weighted pseudo population, and then the difference in the baseline covariates was examined in accordance with the value of the standardized mean difference (SMD). SMD < 0.1 indicated a negligible difference between the two study cohorts. The Kaplan–Meier method was used to estimate the cumulative incidence rate of dementing disorders during the 16-year follow-up period. The log-rank test was conducted to evaluate the differences between the two study groups. The risk of dementing disorders (dementia, AD, or vascular dementia) was evaluated using a univariate Cox proportional hazards regression model that estimates the hazard ratio (HR) and 95% confidence interval (CI). In addition, a univariate Fine–Gray subdistribution hazard competing risk regression model [30, 31] with death as a competing event was used to estimate the subdistribution HR (sHR) and 95% CI of dementing disorders. Statistical analyses were performed using SAS software version 9.4 and Stata version 15. A *p* value of < 0.05 was considered statistically significant.

## Results

During the study period, 7025 NPH patients were identified. After exclusion, 2053 iNPH patients aged ≥ 60 years were enrolled in this study and classified into SSG (n = 376) and NSSG (n = 1677). Before IPTW (Table 1), SSG had a smaller mean age, smaller percentage of patients with age ≥ 80, smaller percentage of patients with monthly income ≤ 15839 new Taiwan dollar (NTD), greater percentage of patients with monthly income 15840–24999 NTD, smaller percentage of patients during the incidence year of 2007–2012, greater percentage of patients during the incidence year of 2013–2018, higher mean CCI score, smaller percentage of patients with coronary artery disease, and higher percentage of patients with hypertension compared with NSSG. Propensity score matching and stabilized IPTW were then conducted to produce two well-balanced pseudo-cohorts across all potential confounders, and this approach reduced selection bias [28, 29]. IPTW resulted in well-balanced groups across all potential confounders (Table 1). After IPTW, the mean age and CCI score were 75.53 ± 7.41 years and 1.85 ± 1.69, respectively, for SSG, and 76.14 ± 7.61 years and 1.83 ± 1.80, respectively, for NSSG. The distributions of other demographic and clinical characteristics, including monthly income, incidence year, coronary artery disease, and hypertension, were also well-balanced.

**Table 1:** Demographic and clinical characteristics of the two study groups before and after stabilized inverse probability of treatment weighting (IPTW)

	Before IPTW			After IPTW		
	Shunt surgery (n = 376)	No-shunt surgery (n = 1677)	SMD	Shunt surgery (n = 375.0)	No-shunt surgery(n = 1677.4)	SMD
Age (year)						
Average	73.69 ± 7.18	76.54 ± 7.62	**0.385**	75.53 ± 7.41	76.14 ± 7.61	0.081
60–69	108 (28.7%)	343 (20.5%)	**0.193**	81 (21.6%)	367 (21.9%)	0.008
70–79	185 (49.2%)	704 (42%)	**0.145**	166 (44.3%)	728 (43.4%)	0.018
80 +	83 (22.1%)	630 (37.6%)	**0.344**	128 (34.1%)	582 (34.7%)	0.012
Sex						
Male	215 (57.2%)	953 (56.8%)	0.007	210 (56.1%)	954 (56.9%)	0.016
Female	161 (42.8%)	724 (43.2%)	0.007	165 (43.9%)	723 (43.1%)	0.016
Monthly income (NTD)						
≤ 15839	59 (15.7%)	405 (24.2%)	**0.213**	83 (22.2%)	379 (22.6%)	0.010
15840–24999	222 (59%)	906 (54%)	**0.101**	209 (55.6%)	922 (55%)	0.013
25000–39999	53 (14.1%)	207 (12.3%)	0.052	47 (12.6%)	213 (12.7%)	0.004
≥ 40000	42 (11.2%)	159 (9.5%)	0.056	36 (9.6%)	164 (9.8%)	0.004
Incidence year						
2001–2006	114 (30.3%)	535 (31.9%)	0.034	119 (31.7%)	531 (31.6%)	0.002
2007–2012	115 (30.6%)	606 (36.1%)	**0.118**	133 (35.5%)	588 (35.1%)	0.009
2013–2018	147 (39.1%)	536 (32%)	**0.150**	123 (32.8%)	558 (33.3%)	0.011
Comorbidity						
CCI score	2.06 ± 1.8	1.78 ± 1.8	**0.155**	1.85 ± 1.69	1.83 ± 1.80	0.011
Coronary artery disease	54 (14.4%)	309 (18.4%)	**0.110**	66 (17.6%)	296 (17.7%)	0.002
Chronic kidney disease	31 (8.2%)	136 (8.1%)	0.005	29 (7.7%)	137 (8.2%)	0.017
Hypertension	261 (69.4%)	1027 (61.2%)	**0.172**	231 (61.6%)	1052 (62.7%)	0.024
Type II diabetes mellitus	105 (27.9%)	499 (29.8%)	0.040	109 (29.1%)	493 (29.4%)	0.007

Continuous variables are presented as mean ± standard deviation. Categorical variables are presented as frequencies and percentages. NTD, new Taiwan dollar. CCI, Charlson comorbidity index. A standardized mean difference (SMD) ≤ 0.1 indicates a negligible difference between the two study groups. SMD > 0.1 is indicated by bold font

**Fig. 2:**
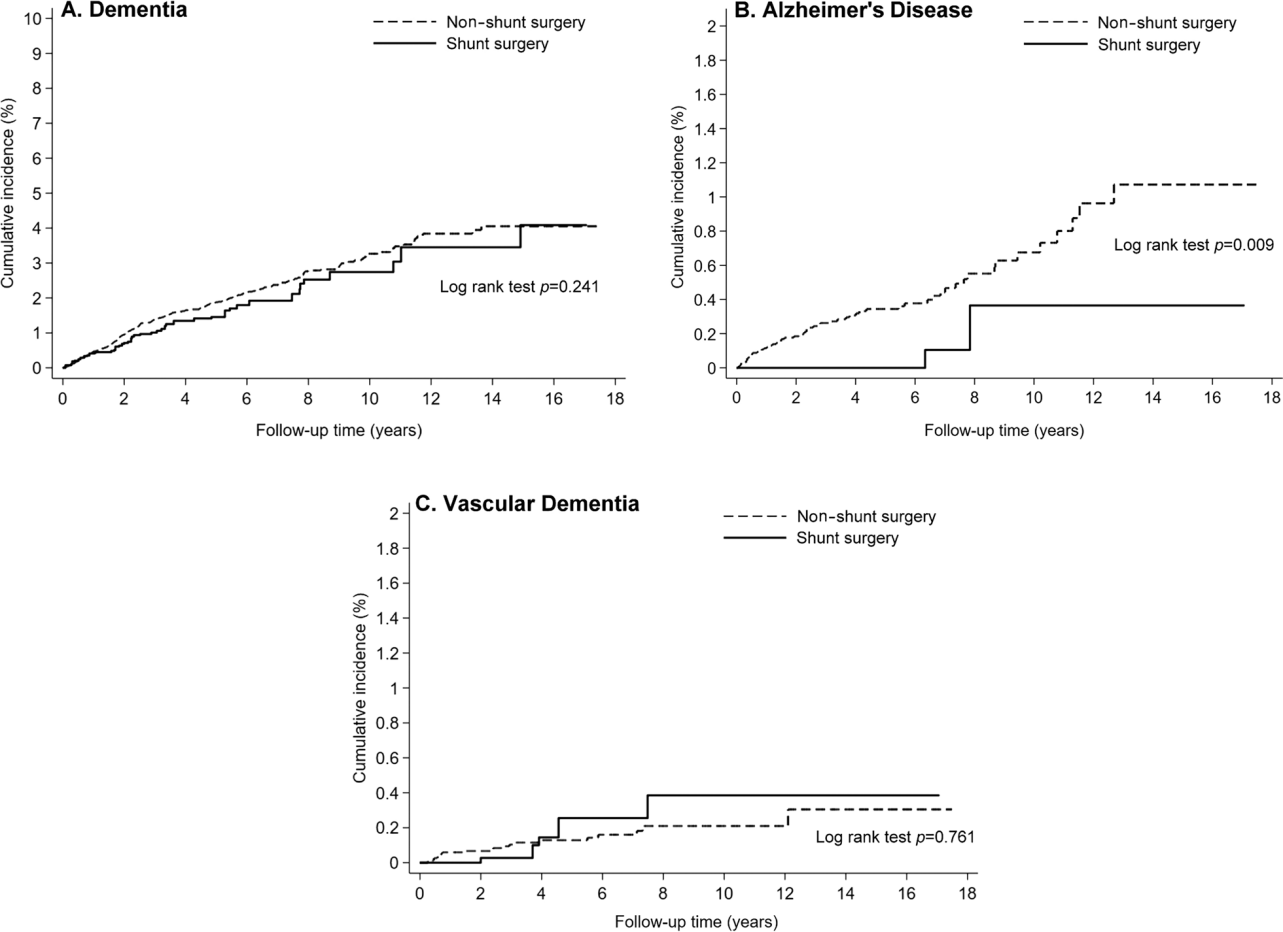
Kaplan–Meier curves showing the cumulative incidence of dementia (**A**), AD (**B**), and vascular dementia (**C**) in patients with iNPH. Patients were classified into two study groups: one group received CSF shunting placement surgery (SSG) and the other did not receive CSF shunting placement surgery (NSSG)

The weighted incidence rates of dementia, AD, and vascular dementia were 34.3, 1.3, and 3.2 per 1000 person-years, respectively, for SSG, and 41.2, 7.7, and 2.6 per 1000 person-years, respectively, for NSSG (Table 2). The Kaplan–Meier curve analysis showed that the cumulative incidence rate of AD (Fig. 2B, log-rank test, *p* = 0.009), but not dementia (Fig. 2A, log-rank test, *p* = 0.241) and vascular dementia (Fig. 2C, log-rank test, *p* = 0.761), in SSG was significantly lower than that in NSSG over the 16-year follow-up period. The average follow-up durations for dementia, AD, and vascular dementia were 4.13 ± 3.60, 4.53 ± 3.70, and 4.57 ± 3.71 years, respectively. The Cox proportional hazards regression analysis revealed that SSG had a reduced HR for developing AD [HR (95% CI) 0.17 (0.04–0.69)], but not for dementia [HR (95% CI) 0.83 (0.61–1.12)] and vascular dementia [HR (95% CI) 1.18 (0.44–3.16)], compared with NSSG (Table 2). Further Fine–Gray subdistribution hazard competing risk regression analysis demonstrated that SSG had a reduced sHR for developing dementia [sHR (95% CI) 0.74 (0.55–0.99)] and AD [sHR (95% CI) 0.15 (0.04–0.61)], but not for vascular dementia [sHR (95% CI) 1.07 (0.40–2.86)], compared with NSSG (Table 2).

**Table 2:** IPTW survival analysis estimating the HR and sHR of dementia, AD, and vascular dementia among patients with iNPH

Outcomes	Number of patients	Event	Person years	Incidence rate†	HR (95% CI)	sHR (95% CI)
Dementia						
Non-shunt surgery	1677.4	294.3	7152.4	41.2	1	1
Shunt surgery	375.0	49.1	1432.1	34.3	0.83 (0.61, 1.12)	0.74 (0.55, 0.99)*
Alzheimer's disease						
Non-shunt surgery	1677.4	60.0	7807.6	7.7	1	1
Shunt surgery	375.0	2.0	1556.8	1.3	0.17 (0.04, 0.69)*	0.15 (0.04, 0.61)*
Vascular dementia						
Non-shunt surgery	1677.4	20.9	7921.3	2.6	1	1
Shunt surgery	375.0	4.9	1550.5	3.2	1.18 (0.44, 3.16)	1.07 (0.40, 2.86)

IPTW, inverse probability of treatment weighting. iNPH, idiopathic normal-pressure hydrocephalus. †, per 1000 person-years. HR, hazard ratio obtained from Cox proportional hazards regression analysis. sHR, subdistribution hazard ratio obtained from Fine–Gray subdistribution hazard competing risk regression analysis. *, *p* < 0.05

## Discussion

The major finding of this population-based propensity-weighted cohort study is that iNPH patients who received CSF shunt surgery exhibited a reduced risk of the subsequent development of dementia and AD compared with those in NSSG. Dementia is a general term for several dementing disorders, and AD is the most common form of dementia [32]. Our finding of reduced risk of dementia was based on the results that demonstrated that SSG exhibited a decrease in sHR. Our finding of reduced risk of AD was based on the results that revealed that SSG presented decreases in the cumulative incidence rate over the 16-year follow-up period, HR, and sHR.

Several pieces of evidence suggest that iNPH patients exhibit a high risk of developing dementing disorders. A prospective study enrolled clinically suspected iNPH patients [13] and found that nearly 60% of these patients became demented, with AD as the most common cause of dementia, followed by vascular dementia, within a median follow-up interval of 4.8 years. The authors also reported that the demented population comprised 73%, 63%, and 46% of non-shunted, shunt-unresponsive, and shunt-responsive patients, respectively. Another study [14] documented that 63% of shunt-responsive iNPH patients had clinical dementia, with AD and vascular dementia as the most common diagnoses, at the end of a median follow-up interval of 4.8 years. A retrospective cohort study [15] found that 21% of shunted iNPH patients developed clinical AD during a median follow-up of 5.3 years. This occurrence was significantly increased compared with that of the general population. Several studies have shown that brain biopsies obtained during CSF shunt surgery in iNPH patients exhibit a high prevalence of typical AD histopathological findings, suggesting pre-stage AD in iNPH patients [16, 17].

A report from the ISHCSF task force [18] indicated that AD and vascular dementia are two common comorbidities of iNPH, and it proposed that iNPH may increase the risk of AD and other dementing disorders. In particular, iNPH and AD have been hypothesized to have a common pathophysiological basis in CSF circulatory dysfunction and failure, and the risk of AD may increase once iNPH takes hold [19]. Collectively, these studies suggest that iNPH patients are predisposed to developing dementing disorders. However, no study has yet compared the risk of developing dementing disorders between shunted and non-shunted patients. Our findings provide evidence to suggest the clinical benefits of CSF shunt surgery in iNPH patients.

Several studies [1, 6–10] have reported that CSF shunt surgery improves the motor (gait disturbance and urinary incontinence) and cognitive disabilities of iNPH patients for the first 2–3 years, but the response rate varies widely among studies. Several clinical predictors have been applied to preoperatively select iNPH patients who can potentially respond to CSF shunt surgery [6, 7, 11]. If iNPH patients undergo CSF shunt surgery, then they will typically need the procedure for the rest of their lives and require regular monitoring. Decision-making typically involves the neurologist, patient, and his/her family members, and it is based on the assessment of the risks and benefits of CSF shunt surgery [11, 12]. Our findings may provide valuable information for assessing the benefit-to-risk profile of CSF shunt surgery in iNPH patients.

The exact reasons why CSF shunt surgery reduces the risk of dementia and AD in iNPH patients remain unclear. The enlarged cerebral ventricles due to excess CSF in iNPH may damage the brain, leading to neurodegenerative diseases [7]. In addition, impaired vascular compliance in iNPH may trigger a cascade of events that lead to irreversible dementia [3]. Furthermore, the accumulation of amyloid due to CSF circulatory dysfunction and failure in iNPH may substantially increase the risk of AD [19]. Theoretically, CSF drainage by shunt implantation in these patients may improve the size of cerebral ventricles [7], vascular compliance [3], and CSF circulatory function [19], leading to reduced risk of dementia and AD.

In the current study, iNPH patients were defined as subjects aged ≥ 60 years and without several known causes for iNPH within 1 year prior to the diagnosis of iNPH. The two original study cohorts (before IPTW) exhibited certain differences in demographic and clinical data, particularly age and comorbidity, which might contribute to the risk of dementia and AD [32]. We then performed stabilized IPTW by using the propensity score to achieve a balanced distribution of confounders across the two study groups and minimize selection bias [28, 29]. The use of stabilized IPTW in the pseudo data preserves the sample size of the original data, produces an appropriate estimation of the variance of the major effect, and estimates HR with less bias compared with other cohort-matching methods [28, 29]. In the current study, we initially used the Kaplan–Meier method and Cox proportional hazards regression model to estimate the cumulative incidence rate during the follow-up period and the risk of dementing disorders, respectively. The two methods are commonly applied to the analysis of survival data, but they do not consider competing risk, which is an event whose occurrence precludes the occurrence of the primary event of interest [31]. To address this issue, we then performed the Fine–Gray subdistribution hazard competing risk regression model, while considering death as a competing event; this method provides direct estimates of absolute risks for developing the primary event of interest [30, 31].

In this study, we identified iNPH using diagnoses G91.0-G91.2. G91.2 is regarded as iNPH, rest being other cases of communicating hydrocephalus. Some clinicians tended to be conservative when giving the diagnosis of iNPH. As such, we pooled diagnoses G91.0-G91.2 together, but excluded patients who had traumatic brain injury, brain tumor, central nervous system infection, spontaneous intracerebral hemorrhage, or stroke with infarction. We sub-analyzed data just for G91.2 and the data are similar to those with pooled diagnoses G91.0-G91.2 (Additional file 2: Table S2).

It should be noted that the study period of this investigation was from 2000 to 2018. As such, the nomenclature and definitions of dementia and AD were based on the Guidelines available during that period [25, 26] and are different from those defined by the updated Guidelines. For example, the National Institute on Aging and Alzheimer’s Association Research Framework suggests that AD is defined by its underlying pathologic processes that can be documented by biological evidence obtained from postmortem or i*n vivo* examination of biomarkers [33]. Clinically diagnosed AD should be referred to as Alzheimer's clinical syndrome, but not as AD or some modified form of AD [33]. Also, the latest edition of the Diagnostic and Statistical Manual of Mental Disorders (DSM-V), a publication by the American Psychiatric Association (APA), recommends that dementia should be referred to as a major neurocognitive disorder, regardless of etiology [34]. Dementia, as the diagnosis, can be used and it should be referred to as a group of symptoms that can accompany certain diseases or conditions, but not a disease itself [34]. Due to these differences in nomenclature and definitions of dementia and AD, findings in this study should be interpreted with caution.

### Strength and limitations

The strength of this work is that it involved a large number of subjects with follow-up over significant periods, and thus, it can be very informative. However, the current study has some limitations. First, our retrospective study was subject to several biases, including data collection and differences between the two study cohorts. We believed that these biases can be minimized via stabilized IPTW. However, unmeasured or inherent differences between the two study cohorts may still account for our observed results. Second, covariates, such as disease severity, smoking, lifestyle, and education, may be related to the development of dementing disorders. Also, detailed medical data are required to comprehend how the diagnosis of iNPH, dementia, AD, or vascular dementia was made. However, the health insurance database used in this study does not register these data. Third, we aimed to explore the risk of developing dementing disorders subsequent to the diagnosis of iNPH, and thus, we excluded patients who were diagnosed with dementia, AD, or vascular dementia prior to the diagnosis of iNPH. For this reason, our findings cannot be generalized to these iNPH patients and the incidence of iNPH may have been underestimated. Moreover, the incidence rate of iNPH reported in this study is believed to be lower than the actual incidence rate. The precise epidemiology of iNPH in our country remains to be defined.

## Conclusions

In conclusion, our results suggest that CSF shunt surgery is associated with reduced risks of the subsequent development of dementia and AD in iNPH patients. Our findings may provide a new perspective for assessing the benefit-to-risk profile of CSF shunt surgery among iNPH patients.

### Supplementary Information

**Additional file 1: Table S1.** ICD-9-CM codes, ICD-10-CM codes, procedure codes, or ATC codes for the diagnosis, surgeries, or drugs searched in this study.

**Additional file 2: Table S2.** IPTW survival analysis estimating the HR and sHR of dementia, AD, and vascular dementia among patients with iNPH (subanalysed data just for G91.2).

## Data Availability

The data generated in this study are available upon request from the corresponding author.

## Abbreviations

iNPH: Idiopathic normal-pressure hydrocephalus
CSF: Cerebrospinal fluid
AD: Alzheimer’s disease
HR: Hazard ratio
sHR: Subdistribution hazard ratio
CI: Confidence interval
NHIRD: National Health Insurance Research Database
ICD-9-CM: International classification of diseases, ninth revision, clinical modification
ICD-10-CM: International classification of diseases, tenth revision, clinical modification
ATC: Anatomical therapeutic chemical
CCI: Charlson comorbidity index
IPTW: Stabilized inverse probability of treatment weighting
SMD: Standardized mean difference
