# Supplementary material for: Cerebrospinal fluid shunt surgery reduces the risk of developing dementia and Alzheimer’s disease in patients with idiopathic normal pressure hydrocephalus: a nationwide population-based propensity-weighted cohort study
Source: Fluids Barriers CNS. 2024 Feb 14;21:16. doi: 10.1186/s12987-024-00517-9 (PMC10868070; doi:10.1186/s12987-024-00517-9)
Supplement: Supplementary file 1 — Additional file 1: Table S1. ICD-9-CM codes, ICD-10-CM codes, procedure codes, or ATC codes for the diagnosis, surgeries, or drugs searched in this study. [file 12987_2024_517_MOESM1_ESM.docx]

**Table S1**. ICD-9-CM codes, ICD-10-CM codes, procedure codes, or ATC codes for the diagnosis, surgeries, or drugs searched in this study.

| Diagnosis | Abbreviation | ICD-9-CM code | ICD-10-CM code |
| --- | --- | --- | --- |
| Communicating hydrocephalus | NPH/iNPH | 331.3 | G91.0-G91.2 |
| Obstructive hydrocephalus |  | 331.4 | G91.1-G91.9 |
| Dementia, mixed, unspecified, senile |  | 331.1-331.9 | G31.01-G31.9 |
| Alzheimer’s disease | AD | 331.0 | G30.0- G30.9 |
| Vascular dementia | VaD | 290.0-290.9  293.90-294.8  310.0-310.9 | F01.50-F05  F06.0-F06.8  F07.0-F07.9, F09 |
| Type II diabetes mellitus | DM | 250.00-250.21 | E08.00-E13.9 |
| Hypertension | HTN | 401.0-401.9  402.0-402.91  403.00-403.91  404.02-404.93  405.01-405.99 | I10  I11.0, I11.9  I12.0, I12.9  I13.0-I13.2  I15.0-I15.9 |
| Chronic kidney (renal) disease | CKD/CRD | 585,593.9 | N18.1- N18.9 |
| Coronary artery disease  Ischemic heart disease | CAD/ IHD | 414.01  414.9  428.0-428.9  429.0-429.3 | I25.110-I25.119  I25.9  I50.1-I50.9,  I51.4-I51.7, I25.10 |
| Traumatic brain injury | TBI | 850.0-850.9  851.00-854.19 | S06.0X0A  S01.90XA, S06.330A, S06.890A, S06.899A |
| Brain tumor (benign or malignant) |  | 225.2, 225.4  225.0-225.9  192.1  191.1-191.9  192.0 | D32.0-D32.9,  D33.0-D33.9,  C70.0-C70.9  C71.0-C71.9,  C72.20-C72.22 |
| Meningitis, central nervous system infection |  | 320.0  320.7  320.9-344.1  326 | G00.0,  G01,  G04.00-G04.91  G09 |
| Spontaneous intracerebral hemorrhage |  | 431  430  432.0-432.9 | I61.0-I61.9  I60.00- I60.9,  I62.00-I62.9 |
| Stroke with infarction |  | 433.00-433.21 | I63.02-I65.29 |

| **Surgery** | **Abbreviation** | **Procedure code** |
| --- | --- | --- |
| Ventriculo-peritoneal shunt | VP shunt | 83049B |
| Lumbao-peritoneal shunt | LP shunt | 83053B |
| CSF test/lumbar CSF external drainage |  | 83054B |
| Revision of CSF shunt |  | 83055B |

| **Drug** | **ATC code** |
| --- | --- |
| Donepezil (Aricept®) | N06DA02 |
| **Rivastigmine (Exelon®)** | N06DA03 |
| Memantine (Witgen**®**) | N06DAX01 |

ICD-9-CM and ICD-10-CM code, International Classification of Diseases, Ninth and Tenth Revision, Clinical Modification; ATC code, Anatomical Therapeutic Chemical code; iNPH, idiopathic normal pressure hydrocephalus; CSF, cerebrospinal fluid.
