# Supplementary material for: Cerebrospinal fluid shunt surgery reduces the risk of developing dementia and Alzheimer’s disease in patients with idiopathic normal pressure hydrocephalus: a nationwide population-based propensity-weighted cohort study
Source: Fluids Barriers CNS. 2024 Feb 14;21:16. doi: 10.1186/s12987-024-00517-9 (PMC10868070; doi:10.1186/s12987-024-00517-9)
Supplement: Supplementary file 2 — Additional file 2: Table S2. IPTW survival analysis estimating the HR and sHR of dementia, AD, and vascular dementia among patients with iNPH (subanalysed data just for G91.2). [file 12987_2024_517_MOESM2_ESM.docx]

**Table S2.** IPTW survival analysis estimating the HR and sHR of dementia, AD, and vascular dementia among patients with iNPH (subanalysed data just for G91.2).

| **Outcomes** | | Number of patients | Event | Person years | Incidence rate† | HR  (95% CI) | sHR  (95% CI) |
| --- | --- | --- | --- | --- | --- | --- | --- |
| **Dementia** | |  |  |  |  |  |  |
|  | Non-shunt surgery | 1524.0 | 288.2 | 7023.9 | 41.0 | 1 | 1 |
|  | Shunt surgery | 325.6 | 46.1 | 1335.4 | 34.5 | 0.83 (0.61, 1.13) | 0.73 (0.54, 1.00) |
| **Alzheimer's disease** | |  |  |  |  |  |  |
|  | Non-shunt surgery | 1524.0 | 55.4 | 7681.2 | 7.2 | 1 | 1 |
|  | Shunt surgery | 325.6 | 2.0 | 1455.3 | 1.4 | 0.19 (0.05, 0.79)* | 0.17 (0.04, 0.68)* |
| **Vascular dementia** | |  |  |  |  |  |  |
|  | Non-shunt surgery | 1524.0 | 21.1 | 7793.2 | 2.7 | 1 | 1 |
|  | Shunt surgery | 325.6 | 5.1 | 1449.1 | 3.5 | 1.26 (0.48, 3.33) | 1.13 (0.43, 2.98) |

IPTW, inverse probability of treatment weighting. iNPH, idiopathic normal-pressure hydrocephalus. †, per 1000 person-years. HR, hazard ratio obtained from Cox proportional hazards regression analysis. sHR, subdistribution hazard ratio obtained from Fine–Gray subdistribution hazard competing risk regression analysis. *, *p* < 0.05.
